# Prevalence of Mental Disorders in Patients With Chronic Thromboembolic Pulmonary Hypertension

**DOI:** 10.3389/fpsyt.2022.821466

**Published:** 2022-03-02

**Authors:** Madelaine-Rachel Dering, Nicole Lepsy, Jan Fuge, Tanja Meltendorf, Marius M. Hoeper, Ivo Heitland, Jan C. Kamp, Da-Hee Park, Manuel J. Richter, Henning Gall, Hossein A. Ghofrani, Dietmar Ellermeier, Hans-Dieter Kulla, Kai G. Kahl, Karen M. Olsson

**Affiliations:** ^1^Department of Psychiatry, Social Psychiatry and Psychotherapy, Hannover Medical School, Hannover, Germany; ^2^Department of Respiratory Medicine, Hannover Medical School, German Center for Lung Research (DZL/BREATH), Hannover, Germany; ^3^Department of Internal Medicine, Justus Liebig University Giessen, Universities of Giessen and Marburg Lung Center, German Center for Lung Research (DZL), Giessen, Germany; ^4^Department of Pneumology, Kerckhoff Heart, Rheuma and Thoracic Center, Universities of Giessen and Marburg Lung Center, German Center for Lung Research, Bad Nauheim, Germany; ^5^Pulmonale Hypertonie Selbsthilfe, Bottrop, Germany; ^6^Pulmonale Hypertonie eV, Rheinstetten, Germany

**Keywords:** chronic thromboembolic pulmonary hypertension, pulmonary hypertension, mental disorder, quality of life, depression disorder, panic disorder, CTEPH

## Abstract

**Objective:**

Pulmonary hypertension (PH) is a chronic and progressive pulmonary vascular disease resulting in symptoms such as shortness of breath and fatigue and leading to death from right heart failure if not adequately treated. Chronic thromboembolic pulmonary hypertension (CTEPH) is a subgroup of PH characterized by obstruction or occlusion of pulmonary arteries by post-embolic fibrotic material. To date, few studies examined symptoms of depression and anxiety in patients with CTEPH, showing depression levels as high as 37.5%. However, none of the former studies used structured expert interviews.

**Methods:**

Mental disorders were diagnosed using the Structured Clinical Interview for DSM-5 (SCID). The prevalence of mental disorders in patients with CTEPH were compared to the prevalence in patients with pulmonary arterial hypertension (PAH) and the general German population. Quality of life (QoL) was measured with World Health Organization (WHO) Quality of Life questionnaire (short form). Factors associated with QoL were analyzed with linear regression and the diagnostic value of the Hospital Anxiety and Depression Scale (HADS) was evaluated using receiver operating characteristics (ROC) curve analysis.

**Results:**

Hundred and seven patients with CTEPH were included. Almost one-third of the patients (31.8%) had current psychological disorders. Panic disorder (8.4%), specific phobia (8.4%), and major depressive disorder (6.5%) were the most prevalent mental illnesses. The prevalence of panic disorders was higher in CTEPH compared to the German population while major depressive disorder was fewer in CTEPH compared to PAH. The presence of mental disorders had a major impact on QoL. Hospital Anxiety and Depression Scale discriminated depression and panic disorder reliably.

**Conclusion:**

Mental disorders are common in patients with CTEPH and associated with an impaired QoL. The HADS may be a useful screening tool for panic and depression disorders in patients with CTEPH. Further research on therapeutic strategies targeting mental disorders in patients with CTEPH is needed.

## Introduction

Chronic thromboembolic pulmonary hypertension (CTEPH) is characterized by non-resolving clots following pulmonary embolism resulting in elevated pulmonary arterial pressure and increased pulmonary vascular resistance (PVR) leading to right heart failure and death if not adequately treated (1). It is categorized as World Health Organization (WHO) Group 4 PH and a debilitating disease with exertional dyspnea and impaired exercise tolerance being the leading symptoms. As in other forms of pulmonary hypertension (PH), patients are prone to develop signs of right-sided heart failure, which is also the leading cause of death in these patients (2–4). The diagnostic and therapeutic options have increased during the last years (5–7). In operable patients, pulmonary endarterectomy (PEA) is the treatment of choice and may be curative whereas inoperable patients can experience symptom relieve by drug therapy and pulmonary balloon angioplasty (BPA) (8, 9).

The diagnosis of CTEPH might impact patients' life beyond the somatic disabilities. The physical impairment might mean a potential inability to participate in everyday life and to continue working, which may result in financial as well as social limitations. In addition, the patients are faced with the outlook of having to undergo invasive procedures and/or suffering from a chronic, life-threatening disease. As a consequence, patients might be at risk of developing concomitant mental disorders.

Previous studies on the prevalence of mental disorders in patients with CTEPH almost exclusively focused on depression and anxiety (10–15). The prevalence of these disorders ranged from 14.3 to 56.5% for depression and 4.1 to 43.5% for anxiety disorders. In a mixed sample of patients with CTEPH and pulmonary arterial hypertension (PAH) evaluated by Zhou et al., the prevalence of depression and/or anxiety was even higher (66.3%) (12). Whereas, all listed studies found impaired quality of life (QoL) in the presence of a mental disorder, the prevalence rates of mental disorders differed substantially and not all studies focused on CTEPH exclusively. Additionally, most studies almost exclusively used self-rating questionnaires rather than structured clinical interviews to determine the presence of mental disorders.

In the present study, we examined the prevalence of current mental disorders, especially depression and anxiety disorders, in patients with CTEPH compared to PAH and the normal population using structured clinical interviews (structured clinical interview for DSM-5, SCID) (16). Additionally, this study aimed to analyze the association between current mental disorders and QoL in CTEPH.

To the best of our knowledge, there has been no comparable study-design for patients with CTEPH yet.

## Methods

### Design and Study Setting

In this observational cross-sectional multicenter study patients ≥18 years with a confirmed diagnosis of CTEPH according to current diagnosis criteria (3, 4, 17–19), who were mentally and physically capable and willing to complete all questionnaires and participate in an interview in German language, were included. The patients were recruited in two large German PH referral centers (Hannover Medical School and University of Gießen and Marburg). Approval of the study was given by the local institutional review boards (Nr. 8540_BO_K_2019 for Hannover and Nr. 21119 for Giessen and Marburg). Written informed consent was given by all included patients. The results presented here are part of a larger examining psychosocial factors in patients with CTEPH. This study focused on the prevalence of mental disorders and its impact on QoL.

### Recruitment

Databases in both centers were searched for active patients with CTEPH.

Contact was established by mail. Written informed consent was obtained from all participants before other study-related procedures were performed.

The structured clinical interviews for DSM-5 were performed by psychologists either face to face or by telephone interview. The decision whether interviews were conducted face to face or by telephone was made depending on pending appointments in the outpatient clinics and the patients' preferences.

### Questionnaires and SCID

All self-rated questionnaires included anthropometric data (age, weight, height, body mass index), lifestyle factors such as sports, smoking habits and current alcohol consumption (drinks per week). The sport score was defined by a 6-point-scale (1 = no sports; 2 = occasionally; 3 = less than one time a week; 4 = one time a week; 5 = more than one time a week; 6 = more than three times a week).

The psychiatric characterization was conducted using the SCID (German translation) for both current and past axis-1 psychiatric disorders. It included a structured set of questions based on diagnostic criteria. Answers given by the interviewed person were rated by the trained interviewer regarding the presence and clinical impact of the symptom. Only current disorders were reported.

The Hospital Anxiety and Depression Scale (HADS) (20) was used to compare diagnostic validity of this frequently used self-rating instrument with the results of expert interviews (SCID). The HADS consists of the two dimensions “anxiety” (HADS-A) and “depression” (HADS-D). Both dimensions contain seven items each that are rated on four-point scales (0–3 points). Results are reported as sum per dimensions. Higher values in this screening tool mean a higher probability of clinically relevant depressive or anxiety disorder.

Quality of life was measured with the WHO QoL questionnaire in short form (WHOQOL-BREF) (21, 22). The WHOQOL-BREF consists of a total of 26 items, each rated on a five-point scale (1–5 points). The questionnaire consists of the scales “physical” (seven items), “psychological” (six items), “social” (three items), and “environment” (eight items). The first two items of the questionnaire are only considered in the calculation of the global health status. The respective scale scores are shown as mean values of the given items. The global health status is shown as the mean value of all items. All scales are converted from 1 to 5 points to 0 to 100 points to ensure comparability with the WHOQOL (23). Higher values in the given scales can be interpreted as higher QoL. We reported four dimensions of the WHOQOL-BREF, i.e., overall, psychological, physical, and social QoL.

The licenses for the German version of the SCID, HADS, and WHOQOL-BREF were acquired. The self-rated questionnaire including anthropometric data and lifestyle factors was self-created.

### Psychiatric Diagnosis

The Diagnostic and Statistical Manual of Mental Disorders, Fifth Edition (DSM-5) was used to define mental disorders (24).

### Clinical Assessments

Clinical assessments included WHO functional class (WHO FC), 6-min walk distance (6MWD), blood gas analysis, lung function tests including diffusion capacity of the lungs for carbon monoxide (DLCO), serum levels of the N-terminal fragment of pro brain natriuretic peptide (NT-proBNP), and body mass index (BMI) at the time of this study. Hemodynamic parameters were documented at diagnosis. All data concerning CTEPH related therapies (medication, BPA, PEA) from diagnosis until psychiatric assessment were collected.

Patients who underwent PEA with no signs of residual PH and no need for medical CTEPH-therapy thereafter were categorized as curatively treated.

### Comparison With Population of Patients With PAH and General Population

The comparison of the prevalence of mental disorders in patients with CTEPH with the prevalence of mental disorders in patients with PAH was conducted using a sample of 217 PAH-patients aged 44–66 years recently described by Olsson et al. (25). Additionally, baseline demographics of the PAH sample were used to compare with those of the CTEPH sample. Data from a sample aged 18–79 years of the mental health module of the German Health Interview and Examination Survey for Adults (DEGS1-MH; *n* = 5,381) (26) were used to compare the prevalence of mental disorders in patients with CTEPH with the prevalence of mental disorders in the general population.

### Statistical Analysis

The analysis of the data was done with the statistical software IBM SPSS Statistics 28.0 (IBM Corp, Armonk, NY, USA) and STATA 13.0 (StataCorp LP, College Station, Texas, USA). Unless indicated otherwise, categorical variables are displayed as *n* and percent (%). Continuous variables are shown as mean and standard deviation (SD) or median and interquartile range (IQR). *t*-Test, Mann-Whitney U-test, or χ^2^-test were used to compare groups, as appropriate. For the comparison of prevalence rates of mental disorders in CTEPH, PAH and a representative German population (DEGS1-MH), χ^2^-tests were used, respectively. Differences between the three populations and point estimates with 95% CIs were calculated. Differences in the prevalence of mental disorders between curatively treated CTEPH patients and non-curatively treated CTEPH patients were analyzed using χ^2^-test. The impact of mental disorders on QoL was examined with linear regression models. Determinants on QoL were calculated with psychological, physical, and overall QoL as dependent variables. The best cut-off values of HADS-A (anxiety) and HADS-D (depression) were calculated using receiver operating characteristics (ROC) curve analysis with calculation of the area under the curve (AUC) and corresponding sensitivity and specificity on the outcomes panic disorder and major depressive disorder derived from the structured clinical interview. All tests were two-sided, statistical significance was considered for *p*-values <0.05.

## Results

### Participant Characteristics

**Figure 1:**
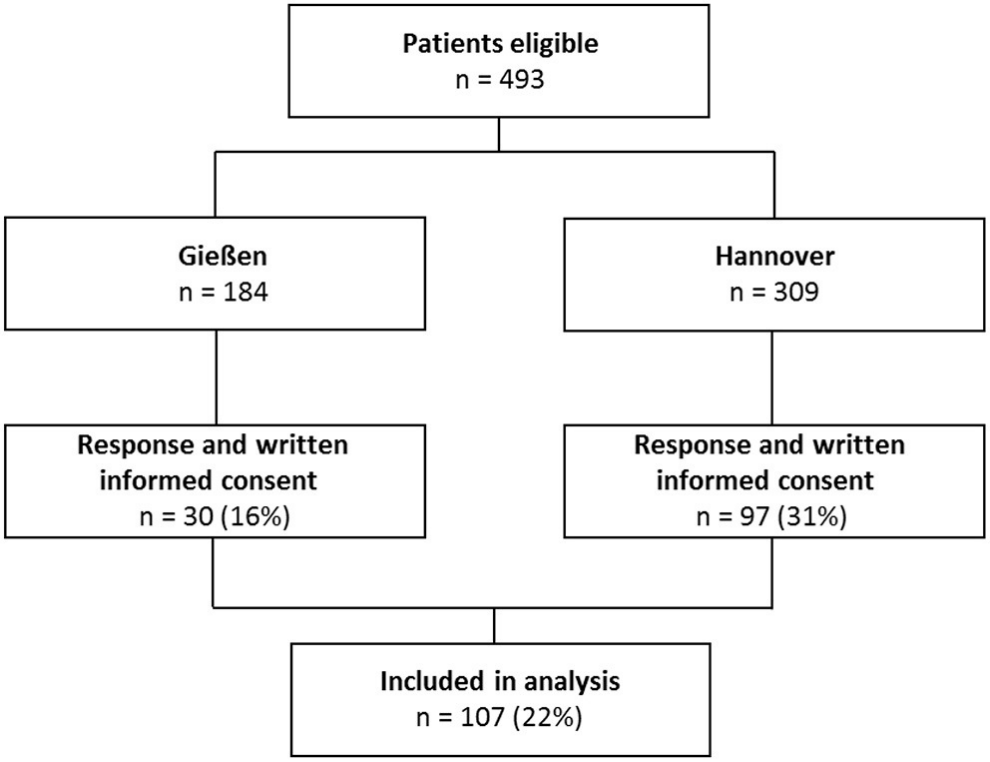
Flowchart of patient inclusion.

All patients were approached and interviewed between December 2019 and May 2021. A total of 493 patients were contacted and 107 patients (22%) finally took part in the interview (Figure 1). Patient's characteristics are shown in Table 1A. Overall, patients with a current mental disorder were younger. In comparison with patients with PAH, the CTEPH sample was significantly older and included more male participants (Table 1B). Time since diagnoses was shorter in patients with CTEPH. Additionally, WHO FC, DLCO, mPAP, PVR, and the number of therapies differed between the groups. There were less active smokers in the CTEPH sample. Also exercise score as well as overall and psychological QoL differed between patients with CTEPH and patients with PAH.

**Table 1A:** Characteristics of the patients at baseline.

	**All patients** ***n* = 107**	**Any current mental disorders** ***n* = 35 (33%)**	**Patients without any current mental disorder** ***n* = 72 (67%)**	***P*** **-value**
Age (years)	69 (56–76)	59 (45–71)	72 (59–77)	**0.001**
Female sex (%)	55 (51%)	18 (51%)	37 (51%)	0.997^b^
BMI (kg/m^2^)	26 (24–31)	26 (24–32)	27 (24–30)	0.348
**Diagnosis**				
History of VTE, *n* (%)	63 (59%)	20 (57%)	43 (60%)	0.854^b^
Time since CTEPH diagnosis (years)^a^	5.4 ± 0.5	4.6 ± 0.8	5.8 ± 0.6	0.208
**WHO FC**				
I, *n* (%)	23 (23%)	7 (21%)	16 (24%)	0.101^b^
II, *n* (%)	33 (31%)	10 (30%)	23 (35%)	
III, *n* (%)	42 (39%)	13 (40%)	27 (41%)	
IV, *n* (%)	3 (3%)	3 (9%)	0 (0%)	
6MWD (m), *n* = 79	449 (337–533)	443 (227–546)	449 (340–519)	0.287
NT-proBNP (ng/l), *n* = 86	168 (69–414)	167 (78–439)	167 (58–421)	0.553
DLCO (% pred.), *n* = 85	62 (53–74)	66 (60–86)	61 (50–71)	0.052
paO_2_, mmHg, *n* = 89	62 (58–70)	63 (60–70)	61 (58–66)	0.285
**Hemodynamics at diagnosis**				
mPAP (mmHg)	42 (33–50)	42 (27–47)	41 (33–52)	0.369
PAWP (mmHg)	9 (6–12)	10 (7–12)	9 (6–12)	0.242
CI (l/min/m^2^)	2.4 (2–2.8)	2.1 (1.8–2.8)	2.4 (2.2–2.8)	0.265
PVR (dyn·s·cm^−5^)	506 (339–743)	485 (316–830)	518 (376–731)	0.873
CTEPH medication^*^				
No therapy, *n* (%)	22 (28%)	9 (36%)	13 (24%)	0.517^b^
Monotherapy, *n* (%)	47 (59%)	13 (52%)	34 (62%)	
Double combination therapy, *n* (%)	11 (14%)	3 (12%)	8 (15%)	
OAC, *n* (%)	101 (94%)	32 (94%)	67 (94%)	0.959^b^
**CTEPH interventions**				
PEA, *n* (%)	27 (25%)	9 (27%)	18 (25%)	0.902^b^
Time since PEA (month)	2 (1–19)	2 (1–6)	2 (1–27)	0.306
BPA, *n* (%)	37 (35%)	14 (42%)	23 (32%)	0.320^b^
BPA sessions	2 (0–5)	2 (0–5)	2 (0–5)	0.932
**Smoking status**				
Active, *n* (%)	2 (2%)	0 (0%)	2 (3%)	0.558^b^
Former, *n* (%)	45 (42%)	14 (40%)	31 (43%)	
Never, *n* (%)	60 (56%)	21 (60%)	39 (54%)	
Packyears	13 (8–26)	12 (8–28)	20 (8–26)	0.805
**Sociodemographic items**				
Drinking (drinks per week)^a^	1.6 ± 0.4	2.6 ± 1.0	1.1 ± 0.3	0.067
Exercise score (points)	3 (2–4)	3 (3–4)	3 (2–4)	0.845^c^
HADS-A (points)	5 (2–9)	8 (4–11)	4 (2–7)	**<0.001**
HADS-D (points)	5 (2–8)	7 (4–10)	4 (1–7)	**0.002**
QoL-overall (points)	72 (65–85)	68 (60–79)	78 (66–90)	**0.002**
QoL-psych (points)	71 (58–79)	58 (46–75)	75 (59–83)	**0.001**
QoL-physical (points)	64 (53–79)	57 (46–71)	71 (55–82)	**0.002**
QoL-social (points)	71 (54–83)	67 (42–83)	75 (58–83)	0.110

*BMI, body mass index; VTE, venous thromboembolism; CTEPH, chronic thromboembolic pulmonary hypertension; WHO FC, World Health Organization Functional Class; 6MWD, six-min walking distance; BNP, brain natriuretic peptide; NT-proBNP, N-terminal fragment of pro-brain natriuretic peptide; DLCO, diffusion capacity of the lung for carbon monoxide; paO_2_, mmHg, arterial pO_2_; mPAP, mean pulmonary arterial pressure; PAWP, pulmonary artery wedge pressure; CI, cardiac index; PVR, pulmonary vascular resistance; OAC, oral anticoagulation; PEA, pulmonary endarterectomy; BPA, pulmonary balloon angioplasty; HADS-A, hospital anxiety and depression scale-anxiety; HADS-D, hospital anxiety and depression scale-depression; QoL, quality of life; psych, psychological; SD, standard deviation; IQR, inter quartile range. Continuous variables are stated as median and interquartile ranges (IQR) and categorical variables are stated as n and percent (%), unless indicated otherwise*.

a *Mean and SD were used because of distribution of the data*.

b *Non-parametric Pearson's chi-squared test was used because of nominal scale of the variable*.

c *Non-parametric Mann-Whitney U-test was used because of ordinal scale of the variable*.

* *No cases for triple combination therapy*.

*Statistically significant values are shown as bold*.

**Table 1B:** Comparison of the characteristics of the CTEPH and PAH patients at baseline.

	**CTEPH** ***n* = 107**	**PAH** ***n* = 217**	***P*** **-value**
Age (years)	69 (56–76)	56 (44–66)	**<0.001**
Female sex (%)	55 (51%)	155 (71%)	**<0.001**
BMI (kg/m^2^)	26 (24–31)	26 (23–31)	0.308
**Diagnosis**			
Time since diagnosis (years)^a^	5.4 ± 0.5	8.1 ± 4.1	**0.002**
**WHO FC**			
I, *n* (%)	23 (23%)	112 (52%)	**0.012**
II, *n* (%)	33 (31%)		
III, *n* (%)	42 (39%)	93 (43%)	
IV, *n* (%)	3 (3%)	10 (5%)	
6MWD (m)	449 (337–533)	439 (353–521)	0.856
NT-proBNP (ng/l)	168 (69–414)	184 (88–517)	0.854
DLCO (% pred.)	62 (53–74)	62 (47–74)	**0.031**
paO_2_, mmHg	62 (58–70)	67 (60–75)	0.079
**Hemodynamics at diagnosis**			
mPAP (mmHg)	42 (33–50)	48 (41–57)	**<0.001**
PAWP (mmHg)	9 (6–12)	9 (6–12)	0.766
CI (l/min/m^2^)	2.4 (2–2.8)	2.4 (2.0–2.9)	0.435
PVR (dyn·s·cm^−5^)	506 (339–743)	707 (501–947)	**<0.001**
**Medication**			
No therapy, *n* (%)	22 (28%)	–	**<0.001**
Monotherapy, *n* (%)	47 (59%)	44 (20%)	
Double combination therapy, *n* (%)	11 (14%)	102 (47%)	
Triple combination therapy, *n* (%)	–	71 (33%)	
OAC, *n* (%)	101 (94%)	–	
**Smoking status**			
Active, *n* (%)	2 (2%)	24 (11%)	**<0.001**
Former, *n* (%)	45 (42%)	31 (14%)	
Never, *n* (%)	60 (56%)	162 (75%)	
Packyears	13 (8–26)	14 (5–25)	0.804
**Sociodemographic items**			
Drinking (drinks per week)^a^	1.6 ± 0.4	0.8 ± 2.0	0.589
Exercise Score (points)	3 (2–4)	3 (2–4)	**0.021**
HADS-A (points)	5 (2–9)	6 (2–9)	0.147
HADS-D (points)	5 (2–8)	5 (2–8)	0.708
QoL-overall (points)	72 (65–85)	50 (38–75)	**<0.001**
QoL-psych (points)	71 (58–79)	71 (58–79)	**0.003**
QoL-physical (points)	64 (53–79)	57 (45–75)	0.553

*CTEPH, chronic thromboembolic pulmonary hypertension; PAH, pulmonary arterial hypertension; BMI, body mass index; WHO FC, World Health Organization Functional Class; 6MWD, six-min walking distance; BNP, brain natriuretic peptide; NT-proBNP, N-terminal fragment of pro-brain natriuretic peptide; DLCO, diffusion capacity of the lung for carbon monoxide; paO_2_, mmHg, arterial pO_2_; mPAP, mean pulmonary arterial pressure; PAWP, pulmonary artery wedge pressure; CI, cardiac index; PVR, pulmonary vascular resistance; OAC, oral anticoagulation; HADS-A, hospital anxiety and depression scale-anxiety; HADS-D, hospital anxiety and depression scale-depression; QoL, quality of life; psych, psychological; SD, standard deviation; IQR, inter quartile range. Continuous variables are stated as median and interquartile ranges (IQR) and categorical variables are stated as n and percent (%), unless indicated otherwise*.

a *Mean and SD were used because of distribution of the data*.

*Statistically significant values are shown as bold*.

### Prevalence of Mental Disorders in CTEPH

Almost one-third of the patients showed current mental disorders (31.8%). Panic disorder and specific phobia (both 8.4%) were the most frequent disorders, followed by major depressive disorder (6.5%) (Table 2A). The prevalence of major depressive disorder was significantly lower in CTEPH compared to PAH (*p* < 0.001) while there was no difference to the general population. Compared to the general population, the prevalence of panic disorder was significantly increased in CTEPH, *p* < 0.001. The prevalence of mental disorders was independent of the CTEPH therapy, i.e. surgical, interventional, or medical therapy (Table 1A). There were no cases of alcohol dependence, schizophrenia, bipolar disorder, agoraphobia without panic disorder, PTSD, anorexia nervosa, and bulimia nervosa in this cohort at all. In addition, there were no differences between male and female patients concerning the prevalence of mental disorders.

**Table 2A:** Prevalence of common mental disorders of CTEPH in comparison to data from PAH and the general German population (DEGS1-MH).

	**CTEPH** ***n* = 107**	**PAH** ***n* = 217**	**DEGS1-MH** ***n* = 5,318**	***P*****-value** **CTEPH vs. PAH**	***P*****-value** **CTEPH vs. DEGS1-MH**
Any current mental disorder	31.8% (23.7–41.1)	38.2 (32.0–44.9)	27.7% (26.3–29.2)	0.254	0.351
Alcohol abuse	1.9% (0.1–0.7)	0.4% (0.08–2)	1.8% (1.4–2.3)	–^a^	–^a^
Alcohol dependence	0% (0–0.3)	1.3% (0.4–3)	3.0% (2.5–3.6)	–^a^	–^a^
Schizophrenia	0% (0–0.3)	0.4% (0.08–2)	2.6% (2.1–3.2)	–^a^	–^a^
Major depressive disorder	6.5% (2.9–13.5)	23% (17–29)	7.7% (6.9–8.6)	**< 0.001**	0.659
Bipolar 1 disorder	0% (0–0.3)	0.4% (0.08–2)	1.0% (0.7–1.4)	–^a^	–^a^
Panic disorder	8.4% (4.5–15.2)	15.2% (11–20)	2.0% (1.6–2.5)	0.087	**< 0.001**
Agoraphobia	0% (0–0.3)	5.9% (3.5–9.9)	4.0% (3.4–4.7)	–^a^	–^a^
Social phobia	3.7% (1.5–9.2)	3.6% (1.8–7.1)	2.7% (2.2–3.4)	0.974	0.366
Generalized anxiety disorder	3.7% (1.5–9.2)	2.7% (1.2–5.8)	2.2% (1.8–2.8)	0.500	0.138
Specific phobia	8.4% (4.5–15.2)	10.6% (7.1–15.4)	10.3% (9.3–11.3)	0.535	0.465
PTSD	0% (0–0.3)	4.1% (2.2–7.7)	2.3% (1.8–2.8)	–^a^	–^a^
Obsessive compulsive disorder	0.9% (0.1–5.1)	5.9% (3.5–9.9)	3.6% (3.1–4.4)	–^a^	–^a^
Anorexia nervosa	0% (0–0.3)	0% (0–1.7)	0.7% (0.5–1.1)	–^a^	–^a^
Bulimia nervosa	0% (0–0.3)	0.4% (0.1–2)	0.2% (0.1–0.3)	–^a^	–^a^

*CI, confidence interval; PTSD, posttraumatic stress disorder; CTEPH, chronic thromboembolic pulmonary hypertension; PAH, pulmonary arterial hypertension; DEGS1-MH, Studie zur Gesundheit Erwachsener in Deutschland (German study of health status in adults)-Mental Health. Psychological disorder prevalence rates per group stated as percent (%) and 95% confidence intervals (CI) and risk ratios*.

a *Samples do not satisfy the standard binomial requirement*.

*Statistically significant values are shown as bold*.

#### Prevalence of Mental Disorders in Curatively vs. Non-curatively Treated CTEPH Patients

In total, 33% (*n* = 9) of all curatively treated patients and 32% (*n* = 25) of all non-curatively treated CTEPH patients were diagnosed with any current mental disorder. Detailed information is provided in Table 2B. Of note, none of the reported findings were significant.

**Table 2B:** Prevalence of current mental disorders in curatively treated in comparison to non-curatively treated CTEPH patients.

	**Curatively treated** ***n* = 27**	**Non-curatively treated** ***n* = 80**	***P*** **-value**
Any current mental disorder	33% (0.2–0.5)	32% (0.2–0.4)	0.902
Alcohol abuse	4% (0–0.2)	1% (0–0.1)	0.416
Major depressive disorder	4% (0–0.2)	8% (0–0.2)	0.482
Panic disorder	4% (0–0.2)	10% (0.1–0.2)	0.308
Social phobia	0 (0–0.1)	5% (0–0.1)	0.236
Generalized anxiety disorder	0 (0–0.1)	5% (0–0.1)	0.236
Specific phobia	15% (0.1–0.3)	6% (0–0.1)	0.166
Obsessive compulsive disorder	4% (0–0.2)	0 (0–0.1)	0.084

*CI, confidence interval; PEA, pulmonary endarterectomy; PH, pulmonary hypertension; CTEPH, chronic thromboembolic pulmonary hypertension; PTSD, posttraumatic stress disorder. Psychological disorder prevalence rates per group stated as percent (%) and 95% confidence intervals (CI). Curative treatment was defined for patients who underwent PEA with no residual PH and no need for medical CTEPH-therapy. There were no cases detected for alcohol abuse, schizophrenia, bipolar disorder, agoraphobia, PTSD, anorexia nervosa, and bulimia nervosa*.

### Diagnostic Value of the Hospital Anxiety and Depression Scale in CTEPH

**Figure 2:**
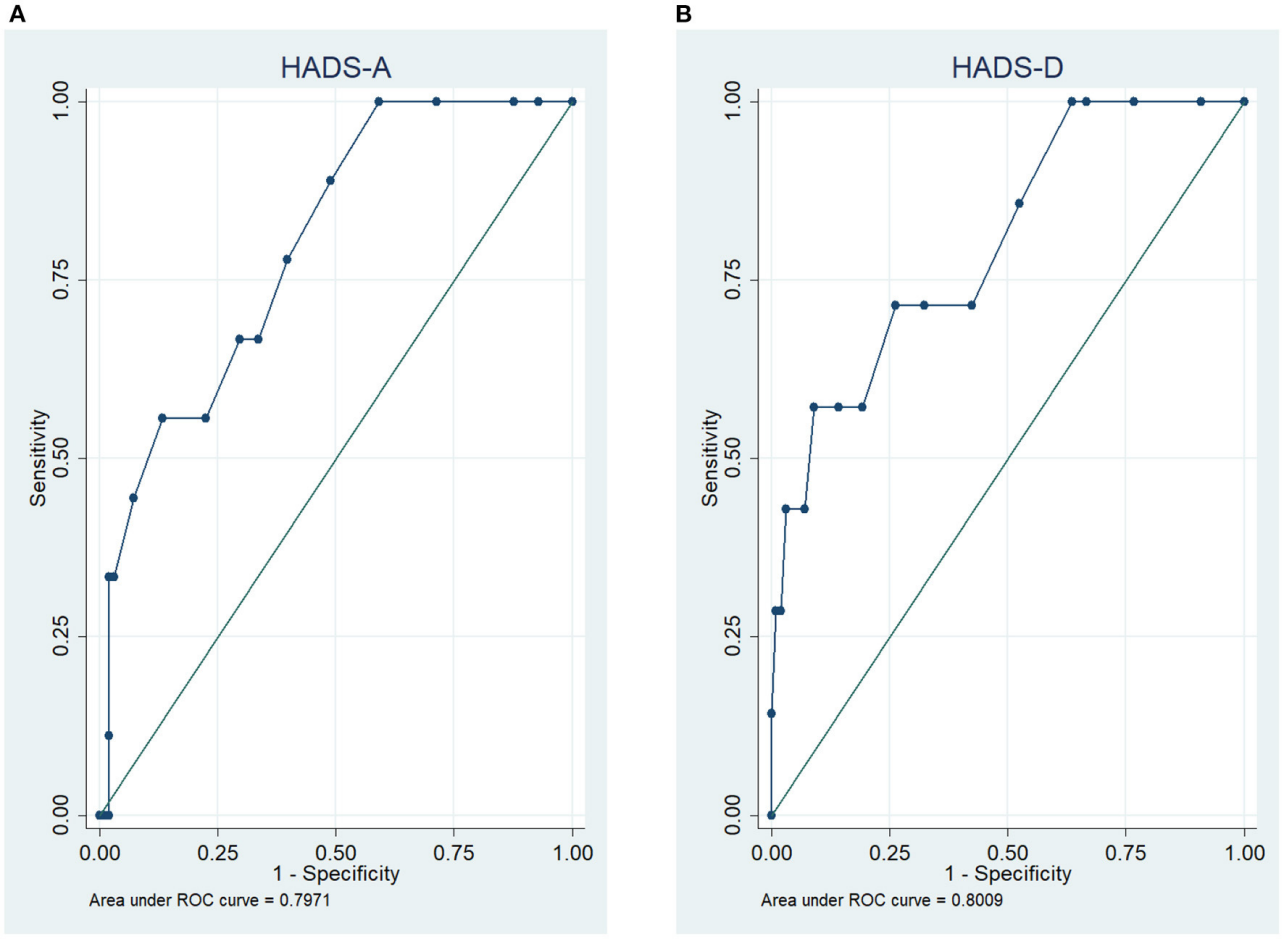
Receiver operating characteristic (ROC) curves of the Hospital Anxiety and Depression Scale (HADS) for the detection of panic disorder **(A)** and depression **(B)**.

The ROC curve analysis of the HADS-D (Depression) showed an AUC of 80.4% (95% CI 63.5–97.3%, *p* = 0.007) and a cut-off value of ≥11 points for the detection of major depressive disorder. Sensitivity was 57.1% and specificity was 91.8% (Figure 2B).

For the HADS-A the ROC curve analysis showed an AUC of 79.7% (95% CI 66.1–93.3%, *p* = 0.003) for the detection of panic disorder at a cut-off value of ≥11 points with a sensitivity of 57.1% and specificity of 90.9% (Figure 2A).

### Quality of Life in CTEPH

For all patients with CTEPH, the overall QoL assessed by the WOHQOL-BREF tool was 72 (65–85) points. Differentiated by presence of mental disorder, the overall QoL for patients with any current mental disorder was significantly lower with 69 (60–79) points compared to patients without any current mental disorder having 78 (66–90) points, *p* = 0.002. Both psychological and physical QoL were significantly lower in patients with any current mental disorder while there were no differences in the severity of CTEPH in the somatic assessment (WHO FC, 6MWD, NT-proBNP) at the time of the psychiatric assessment (Table 1A). The social QoL did not differ between the groups.

### Determinants of QoL in CTEPH

A linear regression analysis distinguished sports score, mental disorder and number of therapies as significant determinants of overall QoL (*r*^2^ = 31.1%), see Table 3. For the sports score, each point-raise was associated with a four-point increase in the overall QoL. Any current mental disorder was associated with a nine-point and each further therapy with a five-point decline in overall QoL. Sports score, current mental disorder and drinks per week were detected as significant determinants for psychological QoL (*r*^2^ = 20.4%). Each increase in sports score was associated with a three-point increase in the psychological QoL. A current mental disorder was associated with a 13-point decrease and drinks per week with a one-point decrease per drink in psychological QoL. World Health Organization functional class was the only significant determinant for physical QoL (*r*^2^ = 31.3%) which was associated with an 11-point decline per each WHO FC increase.

**Table 3:** Results of the linear regression analysis for determinants of quality of life.

	**Coefficient**	***P*** **-value**	**95% CI**
	**Impact on overall QoL**		
Constant	73	**<0.001**	63.6 to 82.6
Sport score	4	0.001	1.5 to 5.8
Mental disorder	−9	0.003	−15.2 to −3.3
Number of therapies	−5	0.013	−9.7 to −1.2
	**Impact on psychological QoL**		
Constant	64	**<0.001**	53.6 to 73.6
Mental disorder	−13	0.001	−20.8 to −5.5
Sport score	3	0.036	0.2 to 5.9
Drinks per Week	−1	0.040	−2.5 to −0.1
	**Impact on physical QoL**		
Constant	91	**<0.001**	78.2 to 100
WHO FC	−11	**<0.001**	−15.9 to −6.4
Mental disorder	−11	0.009	−19.2 to −2.9

*CI, confidence interval; QoL, quality of life; WHO FC, World Health Organization Functional Classification. Coefficients are stated as point values in the associated scales of the World Health Organization Quality of Life Assessment. Statistically significant values are shown as bold*.

## Discussion

In summary, one third of the patients with CTEPH in our cohort had a current mental disorder, similar to what has been reported in the general population or patients with PAH. In patients with CTEPH, the most frequent mental disorders were panic disorder and specific phobia followed by major depression. The presence of any mental disorder had a major negative impact on overall and psychological QoL. Moreover, the HADS was identified as a possible screening tool for the identification of possibly present panic disorder and major depressive disorder in patients with CTEPH.

The prevalence of panic disorder in this study was comparable to previous findings in patients with other forms of PH (10–12, 14). One reason for the significantly increased prevalence of panic disorders in patients with CTEPH compared to the general population might be that symptoms of dyspnea can be perceived as threatening, cause anxiety, and potentially leading to avoidance of potentially dyspnea evoking activities (27). If this is linked to the actual perceived threatening experience of dyspnea connected to CTEPH in those patients, this could ensure that the mere appearance of the symptom leads to a negative evaluation which in turn triggers anxiety. Rumination due to breathlessness has already been described in CTEPH and has been associated with psychological distress (13).

Compared to PAH, the prevalence of panic disorder in CTEPH tended to be lower although this difference did not reach statistical significance. Most previous studies concerning anxiety disorders in CTEPH compared to PAH have yielded different results (10, 11, 14). Lower rates of anxiety disorders in patients with CTEPH compared to patients with PAH might be explained by the availability of more effective, sometimes even curative, therapeutic options for CTEPH such as PEA surgery and balloon pulmonary angioplasty (17, 28–30). At the same time there is no comparable curative treatment available for patients with PAH except lung transplantation and survival is worse despite emerging therapies (19, 31).

The prevalence of specific phobia (anxiety disorder characterized by severe, unreasonable and irrational fear with a specific situation or object) (24, 32) in CTEPH did not differ from the prevalence in the general population or in patients with PAH. This result is consistent with the assumption that specific phobias have a moderate heritability (33). The presence of a chronic disease might therefore have a rather small impact on its prevalence.

In contrast to most previous studies, major depression was less frequent compared to patients with PAH and comparable to the general population (10–12, 14, 15). As in anxiety disorders, we assume that the better treatment opportunities and better prognosis in CTEPH might explain these findings. Additionally, the most commonly used questionnaire in the above mentioned studies (11–13) was the Patient Health Questionnaire 9 (PHQ-9) (34), which has already been found to overestimate the prevalence of major depression compared to SCID (35). Another explanation may be that in two studies in which the HADS was used, the cut-off value for depressive disorders was eight points (10, 14). In our study, a cut-off value of 11 points was used, which was calculated as described. Nevertheless, the high prevalence of major depression in previous studies is of importance given the strong negative impact of CTEPH on mental wellbeing.

Examining the prevalence of mental illnesses in “curatively treated patients” vs. non-curatively treated CTEPH patients, a numerically higher proportion of major depressive disorder, panic disorder, generalized anxiety disorder, and social phobia was found in the latter, whereas the prevalence of specific phobia and obsessive-compulsive disorder tended to be higher in curatively treated patients. Although none of these differences were statistically significant and based on small sample sizes, these observations might be of importance for further research specifically addressing the prevalence of mental disorders in curatively vs. non-curatively treated CTEPH patients.

The practical implications of our study need to be further investigated. Since the presence of any mental disorder had a substantial impact on overall and psychological QoL there is a need for screening tools as most PH physicians are not trained in detecting mental disorders. The HADS may be useful for cardiologists and pneumologists to detect hints of panic or major depressive disorder. The comparably high cut-off value for depression in this study is not in line with previously used cut-off values in general medical patients (HADS-A and HADS-D) (36) or adults with congenital heart disease, as another chronic disease (HADS-D) (37) but contributes to a higher specificity. To further examine whether patients with CTEPH who meet the cut-off values in the HADS truly suffer from mental disorders, streamlined access to psychological evaluation and counseling is needed. Psychological counseling might also be of help in decision making especially for CTEPH patients facing surgical therapy. As the presence of a mental disorder may also be associated with worse exercise tolerance and outcome in patients with CTEPH, it would be justified to provide integrated support (38). Overall, an integrative low-threshold treatment could possibly be of benefit for patients with CTEPH.

Besides current mental disorders, an increasing number of CTEPH therapies, increasing WHO FC and drinks per week had a negative impact on QoL. An increase in the frequency of sport was associated with an increase in QoL. Since all of those factors are modifiers of any mental disorder, mutual support (e.g., providing psychological or psychosomatic counseling) could increase the QoL of patients with CTEPH.

There are strengths and limitations to this study. This study focused specifically on patients with CTEPH and used structured face-to-face or telephonic interviews to identify mental disorders. The main limitations are the inclusion of data from only two German referral centers, a possible confounding lack of selected clinical data, and a lack of detailed information on mental status pre-therapy. The most important limitations are probably the relatively small sample size and the response rate of only 22% of the approached patients. Several reasons might have contributed to the low response rate: (1) Lack of general possibility to be personally contacted by their attending PH physician before receiving the questionnaires, (2) a relatively high age in the CTEPH population, (3) the fact that many patients had undergone surgery at the referring centers with follow-up by their local PH physicians and those patients might have been less motivated to participate in our study. The sample therefore might be biased by selecting a subset of patients with inoperable CTEPH or persistent symptomatic PH after surgery, potentially leading to a higher prevalence of mental disorders due to more severe physical disease. It is possible that this bias might have led to an overestimation of current mental disorders in the present study.

Future studies should include the prevalence of mental disorders before the diagnosis of CTEPH. The investigation of social support and physical comorbidities as influencing variables could also be of interest in further studies. The latter was not included in this study while social support was assessed with the WHOQOL-BREF subscale “social” and did not differ between the groups. A more detailed survey of social support could therefore be of benefit. Generally, future studies addressing diagnostic tools and therapeutic interventions are needed to further improve the treatment of patients with CTEPH.

In conclusion, our study showed that almost one third of patients with CTEPH suffer from mental disorders. Panic disorders, specific phobia, and major depression were the most frequent disorders. The presence of any current mental disorder contributed to a decrease of QoL. The HADS seemed to be a useful screening tool for detection of panic disorders and major depressive disorders.

## Data Availability Statement

The raw data supporting the conclusions of this article will be made available by the authors, without undue reservation.

## Ethics Statement

The studies involving human participants were reviewed and approved by Ethic Committee, Hannover Medical School, Hannover, Germany and Ethic Committee, University Medical Center Gießen and Marburg, Marburg, Germany. The patients/participants provided their written informed consent to participate in this study.

## Author Contributions

M-RD and NL were responsible for study design, implementation of the study, data collection, conducting the interviews, statistical analysis, data interpretation, and drafting the manuscript. JF was responsible for study design, implementation of the study, data collection, statistical analysis, data interpretation, and drafting the manuscript. TM, IH, D-HP, HAG, DE, and H-DK were responsible for study design, data interpretation, and revising the manuscript. MH, KK, and KO were responsible for study design, implementation of the study, statistical analysis, data interpretation, and drafting the manuscript. JK, MR, and HG were responsible for study design, implementation of the study, data interpretation, and revising the manuscript. All authors contributed to the article and approved the submitted version.

## Conflict of Interest

MH has received honoraria for lectures and/or consultations from Acceleron, Actelion, Bayer, GSK, Janssen, MSD, and Pfizer, all outside the present study. D-HP has received honoraria for lectures and/or consultations from Janssen. HG has received personal fees from Actelion, personal fees from AstraZeneca, personal fees from Bayer, personal fees from BMS, personal fees from GSK, personal fees from Janssen-Cilag, personal fees from Lilly, personal fees from MSD, personal fees from Novartis, personal fees from OMT, personal fees from Pfizer, personal fees from United Therapeutics, outside the submitted work. HAG has received fees from Actelion, Bayer, Gilead, GSK, MSD, Pfizer, and United Therapeutics, outside the present work. KK has received honoraria for consultations and/or lectures from Eli Lilly, Janssen, Lundbeck, Neuraxpharm, Otsuka, Pfizer, Servier, Schwabe, Takeda, and Trommsdorff/Ferrer, Alexion, and CannaXan advisory board. KO has received honoraria for lectures and/or consultations from Acceleron, Actelion, Bayer, GSK, Janssen, MSD, United Therapeutics and Pfizer, all outside the present study. H-DK was employed by Pulmonale Hypertonie eV. The remaining authors declare that the research was conducted in the absence of any commercial or financial relationships that could be construed as a potential conflict of interest.

## Publisher's Note

All claims expressed in this article are solely those of the authors and do not necessarily represent those of their affiliated organizations, or those of the publisher, the editors and the reviewers. Any product that may be evaluated in this article, or claim that may be made by its manufacturer, is not guaranteed or endorsed by the publisher.
